# Early-life galacto-oligosaccharides supplementation alleviates the small intestinal oxidative stress and dysfunction of lipopolysaccharide-challenged suckling piglets

**DOI:** 10.1186/s40104-022-00711-5

**Published:** 2022-06-03

**Authors:** Shiyi Tian, Jue Wang, Ren Gao, Jing Wang, Weiyun Zhu

**Affiliations:** Laboratory of Gastrointestinal Microbiology, Jiangsu Key Laboratory of Gastrointestinal Nutrition and Animal Health, National Center for International Research on Animal Gut Nutrition, National Experimental Teaching Demonstration Center of Animal Science, College of Animal Science and Technology, Nanjing Agricultural University, Nanjing, 210095 China

**Keywords:** Early-life, Galacto-oligosaccharides, Lipopolysaccharide, Oxidative stress, Suckling piglets

## Abstract

**Background:**

Galacto-oligosaccharides (GOS) are non-digestible food ingredients that promote the growth of beneficial bacteria in the gut. This study investigated the protective effect of the early-life GOS supplement on the piglets’ gut function against the oxidative stress induced by lipopolysaccharide (LPS)-challenge.

**Methods:**

Eighteen neonatal piglets were assigned to three groups including CON, LPS and LPS + GOS groups. The piglets in CON group and LPS group received physiological saline, while those in LPS + GOS group received GOS solution for 13 d after birth. On d 14, the piglets in LPS group and LPS + GOS group were injected with LPS solutions, while the piglets in CON group were injected with the same volume of physiological saline.

**Results:**

The results showed that the early-life GOS supplement blocked the LPS-induced reactive oxygen species (ROS) secretion, malondialdehyde (MDA) production and the increase of pro-apoptotic factor expression. Meanwhile, the early-life GOS supplement improved the activities of antioxidant enzymes, disaccharidase enzymes activities, and digestive enzymes activities, and increased the mRNA abundance of the gene related to nutrient digestion and absorption and the relative protein expression of tight junction. The study also showed that the early-life GOS supplement improved the expression of Hemeoxygenase-1 (HO-1) and NAD(P)H/quinone acceptor oxidoreductase-1 (NQO-1), and activated the AMP-activated protein kinase (AMPK).

**Conclusions:**

These results suggested that GOS enhanced the gut function, reduced the ROS production and pro-apoptotic factors gene expression, and activated the AMPK signaling pathway in LPS-challenged piglets.

**Supplementary Information:**

The online version contains supplementary material available at 10.1186/s40104-022-00711-5.

## Introduction

Newborn piglets are transferred from a relatively sterile and hypoxic placenta to an ambient bacterial and oxygen-rich environment, and are very vulnerable to free radical oxidative damage and pathogen invasion [1, 2]. The transition may cause the accumulation of reactive oxygen species (ROS) in piglet intestines [3]. The suckling piglets are frequently susceptible to the intestinal oxidative stress because their intestines are different from the adult pig intestine, with limited ROS elimination capacity of the immature gut antioxidant defense system [3]. Several studies have been performed to reduce occurrence of oxidative stress by improving gut antioxidant function using different nutritional administrations [4, 5].

Galacto-oligosaccharides (GOS), a common prebiotic supplement, have demonstrated great benefit to gut health [6]. Previous studies well documented that GOS improved the gut function and altered the bacterial composition in piglets [7–9]. Other studies have demonstrated that GOS decreased ROS production in meat of finished pigs and in IPEC-J2 cells, and improved the antioxidant capacity of weaning piglets [10–12]. Moreover, our recent study has found that the early-life GOS supplement enriched the endogenous antioxidants and improved the antioxidant capacity of mitochondria in the liver of suckling piglets [13]. Although our data suggested a positive effect on hepatic antioxidant capability in suckling piglets, whether the GOS supplementation has a protective effect on the gut oxidative stress of suckling piglets needs a further investigation.

Lipopolysaccharide (LPS) is a component derived from the outer membrane of gram-negative bacteria. The exposure of different tissues to extracellular LPS induces a variety of pathophysiological effect on the host, including immune responses, endotoxic shock and tissue injury [14]. Of note, LPS can induce ROS accumulation in tissues through an increased production of ROS intermediates, such as superoxide radicals, lipid peroxides, and nitric oxides [15]. Previous studies demonstrated that the LPS-stimulated piglets had a damaged host antioxidant system and an impaired intestinal integrity [16]. Meanwhile, the imbalanced antioxidant system dysregulates the proliferation, differentiation, and apoptosis of intestinal epithelial cells, causing intestinal inflammation and other diseases [17, 18]. Nowadays, LPS-challenge is a common strategy to construct a gut oxidative stress model of piglets. Here, we hypothesize that the early-life GOS supplementation could alleviate the small intestinal oxidative stress and dysfunction of LPS-challenged suckling piglets. Thus, the ROS production, antioxidant enzymes activities, intestinal morphology, digestive and absorptive capacity, barrier function, and apoptosis-related genes expression of small intestine were evaluated in this study. Hemeoxygenase-1 (HO-1) and NAD(P)H/quinone acceptor oxidoreductase-1 (NQO-1) are nuclear factor (erythroid-derived-2)-like 2 (Nrf2)-mediated phase II metabolizing enzymes, which have antioxidative properties [19]. In addition, recent studies showed that AMP-activated protein kinase (AMPK) activation mediated Nrf2 activation [20–22]. Therefore, we also investigated the level of HO-1, NQO-1 and the phosphorylation level of AMPK to further reveal the underlying mechanism.

## Materials and methods

### Animals, diets and experimental design

Eighteen newborn piglets (Landrace × Duroc × Yorkshire) with an initial birth weight of 1.57 ± 0.04 kg were obtained from 2 sows (9 piglets per litter) with the similar parity (3 or 4 parities). The piglets were housed with their own mothers. The piglets in each litter were assigned to three groups of three piglets, which were the control (CON) group, the control group challenge with LPS (LPS) or the GOS group challenge with LPS (LPS + GOS). The composition of GOS (Quantum Hi-Tech Biological Co., Ltd., China) are as the followings: dry matter content of 95.94%, of which 13.9% was GOS (DP = 5), 23.0% was GOS (DP = 4), 38.2% was GOS (DP = 3), 15.0% was GOS (DP = 2), 8.0% was lactose, 1.3% was glucose, and 0.6% was galactose [9]. The GOS powder was dissolved in physiological saline to prepare GOS solution with 0.5 g/mL concentration. Before GOS was dissolved in physiological saline, the physiological saline was placed in a hot water bath until the temperature of physiological saline was approximately 37 °C. During 13 d after birth, all piglets in the LPS + GOS group were orally administered GOS solution (1 g GOS/kg body weight [8, 23]) per day. Meanwhile, all piglets in the CON group and the LPS group were orally administered the same volume of physiological saline. The solution was infused into each piglet’s mouth by a sterile injector without a needle. On d 14, the piglets in LPS group and LPS + GOS group were intraperitoneally injected with LPS (*Escherichia coli* O55:B5, Sigma-Aldrich) solution of 80 μg/kg body weight [24], while the piglets in CON group were intraperitoneally injected with the same volume of physiological saline. The piglets had free access to sow milk and water. The piglets were individually weighed on d 1, 3, 5, 7, 10 and 14 to control the dose of GOS at 1 g/kg body weight. Health status was monitored daily until 14 days of age, and all piglets remained healthy during the experimental period.

Two hours [24, 25] after the injection with LPS or saline, two 5-mL tubes of blood from the anterior vena cava were collected. And one tube was supplemented with heparin sodium. The blood sample was centrifuged at 3000 × *g* for 15 min at 4 °C for obtaining serum and plasma samples, and then immediately stored at − 80 °C for further analysis. Then, all piglets were euthanized. Mucosal samples and content samples from the proximal duodenum, proximal jejunum and distal ileum were collected, and stored at − 80 °C for the further analysis.

### Intestinal morphology

After separating the small intestine (SI), the length of the SI and the wet weight of the SI were measured. Then, the proximal duodenum, proximal jejunum and distal ileum were preserved in a 4% paraformaldehyde solution, and then stained with hematoxylin and eosin (HE).

### mRNA expression analysis

The total RNA was isolated with Trizol Reagent According to the manufacture’s recommendations (Vazyme Biotech, Nanjing, China). After standardized to 100 ng/μL, the total RNA was reverse-transcribed to cDNA using a HiScript * III RT SuperMix for qPCR reagent kit (Vazyme Biotech, Nanjing, China). The primers are listed in Table S1. The RT-PCR reactions were performed as the previously described [8]. The mRNA expression levels were calculated by the 2^-ΔΔCt^ method [26] and normalized to housekeeping gene *GAPDH*. The housekeeping gene *GAPDH* was selected as the previously described [8].

### Diamine oxidase

According to the instructions of the manufacturer, the levels of diamine oxidase (DAO) in plasma, duodenal mucosa, jejunal mucosa and ileal mucosa were determined with a DAO assay kit (Nanjing Jiancheng Technology Co., Ltd., Nanjing, China).

### Disaccharidases enzyme activity and digestive enzymes activity

The activity levels of the absorptive enzyme (lactase, sucrase and maltase) in brush border and the digestive enzymes (amylase, lipase and chymotrypsin) in intestinal content (Nanjing Jiancheng Technology Co., Ltd., Nanjing, China) were determined according to the manufacturer’s recommendations.

### Antioxidant/oxidant indices analysis

The levels of ROS, malondialdehyde (MDA, thiobarbituric acid (TBA) method), total anti-oxidation capacity (T-AOC), glutathione peroxidase (GSH-Px) and superoxide dismutase (SOD) in plasma, jejunal mucosa and ileal mucosa were measured according to the manufacturer’s instructions (Nanjing Jiancheng Technology Co., Ltd., Nanjing, China).

### Immunoblotting

The total protein of mucosa was extracted with RIPA buffer (Future Scientific Innovation, Nanjing, China) with a protease inhibitor and a phosphatase inhibitor (Beyotime Institute of Biotechnology, Shanghai, China). Then, a standard bicinchoninic acid (BCA) protein assay (Biosharp life science, Hefei, China) was used to measure the protein concentration. The standardized lysates were separated using a 12% SDS–PAGE followed by electro-transferring onto PVDF membranes (Merck Millipore). After incubated in a skim milk TBS buffer, the membranes were incubated with a primary antibody overnight at 4 °C (zonula occludens-1 (ZO-1), Proteintech, 1:1000; Occludin, Proteintech, 1:1000; Claudin-1, Proteintech, 1:500; AMPK, Cell Signaling Technology, 1:1000; p-AMPK, Cell Signaling Technology, 1:1000; HO-1, Proteintech, 1:1000; NQO-1, Proteintech, 1:1000; β-actin, Cell Signaling Technology, 1:1000). After rinse, the membranes were incubated with an anti-rabbit or anti-mouse IgG HRP-conjugated secondary antibody (1:1000; Cell Signaling Technology) for 2 h at room temperature. Immunoblots were analyzed using an electrochemiluminescence system (Tanon, Shanghai, China). Intensities of band were calculated using ImageJ version 1.47 software. Image intensity of part of bands (Claudin-1, Occludin, ZO-1, HO-1 and NQO-1) was normalized to β-actin bands, the image intensity of the p-AMPK was normalized to AMPK band. All data were expressed as a ratio of the control, which was set at 1.

### Statistical analysis

For the data of the growth performance, the model included treatment (CON, LPS or LPS + GOS), age, and the interactive effects of treatment and age as the fixed effects, with pig identification as the random effects. The data of the growth performance were evaluated by two-way ANOVA. When a significant interaction between treatment and age was observed, the data were further analyzed by using one-way ANOVA with Tukey’s post hoc test. For the other data, the model included CON, LPS and LPS + GOS as the fixed effects, with pig identification as the random effects. The individual was the experimental unit for all analyses. And then these data were evaluated by one-way ANOVA with Tukey’s post hoc test. For all data, the statistically significant was considered significant at *P* < 0.05. Results were expressed as means with standard deviations (SD).

## Results

### Growth performance and intestinal growth parameters

**Fig. 1:**
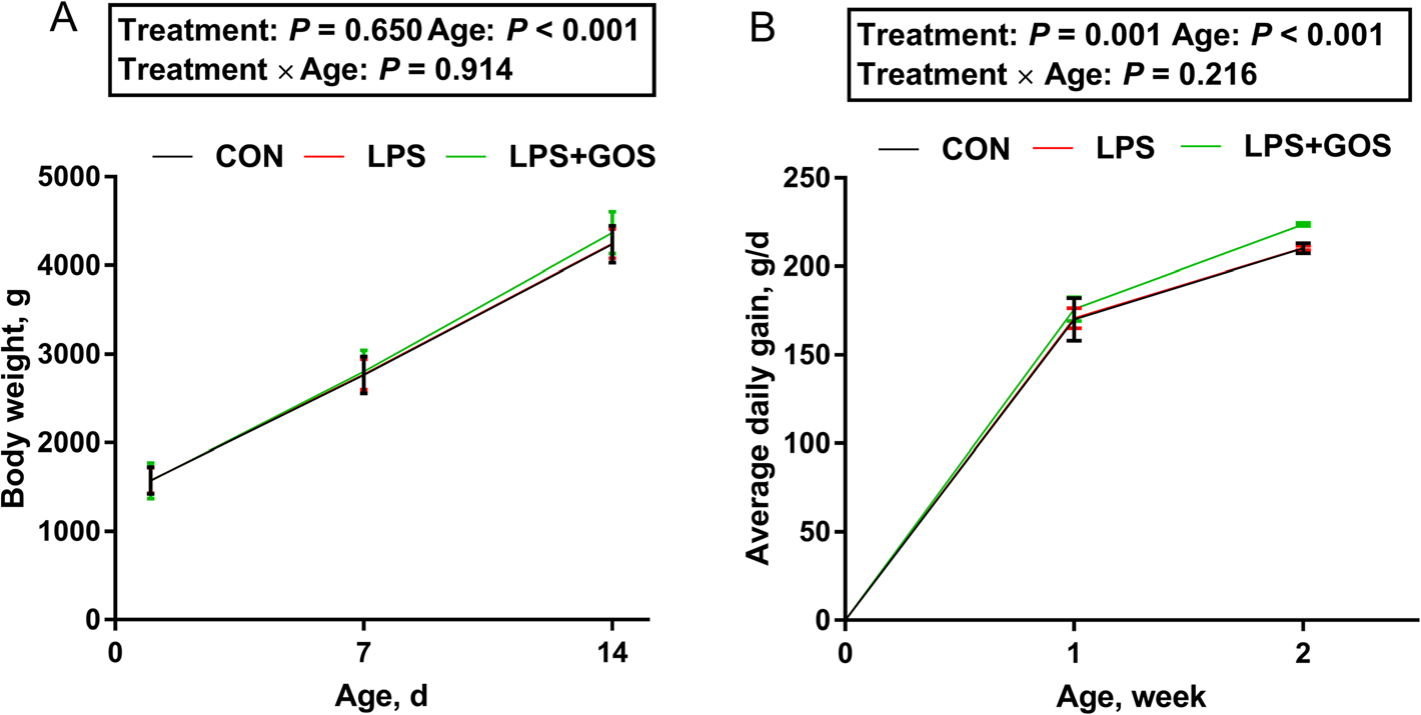
Effects of early-life galacto-oligosaccharides (GOS) supplement on growth performance of LPS-challenged piglets. Piglets were assigned to CON (*n* = 6), LPS (*n* = 6) and LPS + GOS (*n* = 6) receiving physiological saline, physiological saline and GOS solution for 13 d after birth, respectively. On d 14, the piglets in LPS and LPS + GOS group were injected with LPS solution of 80 μg/kg BW, while the piglets in CON group were intraperitoneally injected with the same volume of physiological saline. (**A**) Body weight, (**B**) Average daily gain of suckling piglets. Significant differences (*P* < 0.05) among different treatment piglets within each age are indicated by different letters. Data are expressed as means ± SD

As shown in Fig. 1, treatment and age had no significant interactive effect on the body weight and average daily gain (ADG) of piglets. But a significant treatment effect on ADG of piglets was found (*P* < 0.05). In addition, the results of the digestive organs (Table 1) showed that the intestinal growth parameters of piglets were significantly affected by GOS treatment, showing that the SI weight and SI length were significantly increased compared with the control group and LPS group (*P* < 0.05). Although small intestinal length/body weight was significant in one-way ANOVA, it was not significant in post hoc tests.

**Table 1:** Effects of galacto-oligosaccharides (GOS) on the intestinal growth parameters in LPS-challenged piglets (*n* = 6)

Item	CON	LPS	LPS + GOS	*P* value
SI^1^ weight, g	137.02 ± 2.74^b^	138.35 ± 2.58^b^	146.85 ± 4.79^a^	< 0.001
SI length, m	6.32 ± 0.16^b^	6.33 ± 0.15^b^	6.88 ± 0.24^a^	< 0.001
SI weight/SI length, g/m	21.70 ± 0.62	21.88 ± 0.61	21.34 ± 0.24	0.221
SI weight/body weight, g/kg	32.37 ± 1.27	32.67 ± 1.60	32.66 ± 0.82	0.217
SI length/body weight, m/kg	1.49 ± 0.07	1.49 ± 0.06	1.58 ± 0.03	0.031

^1^*SI* Small intestine

^a,b^Values in the same row with different superscripts are significantly different (*P* < 0.05)

### Antioxidant capacity

**Fig. 2:**
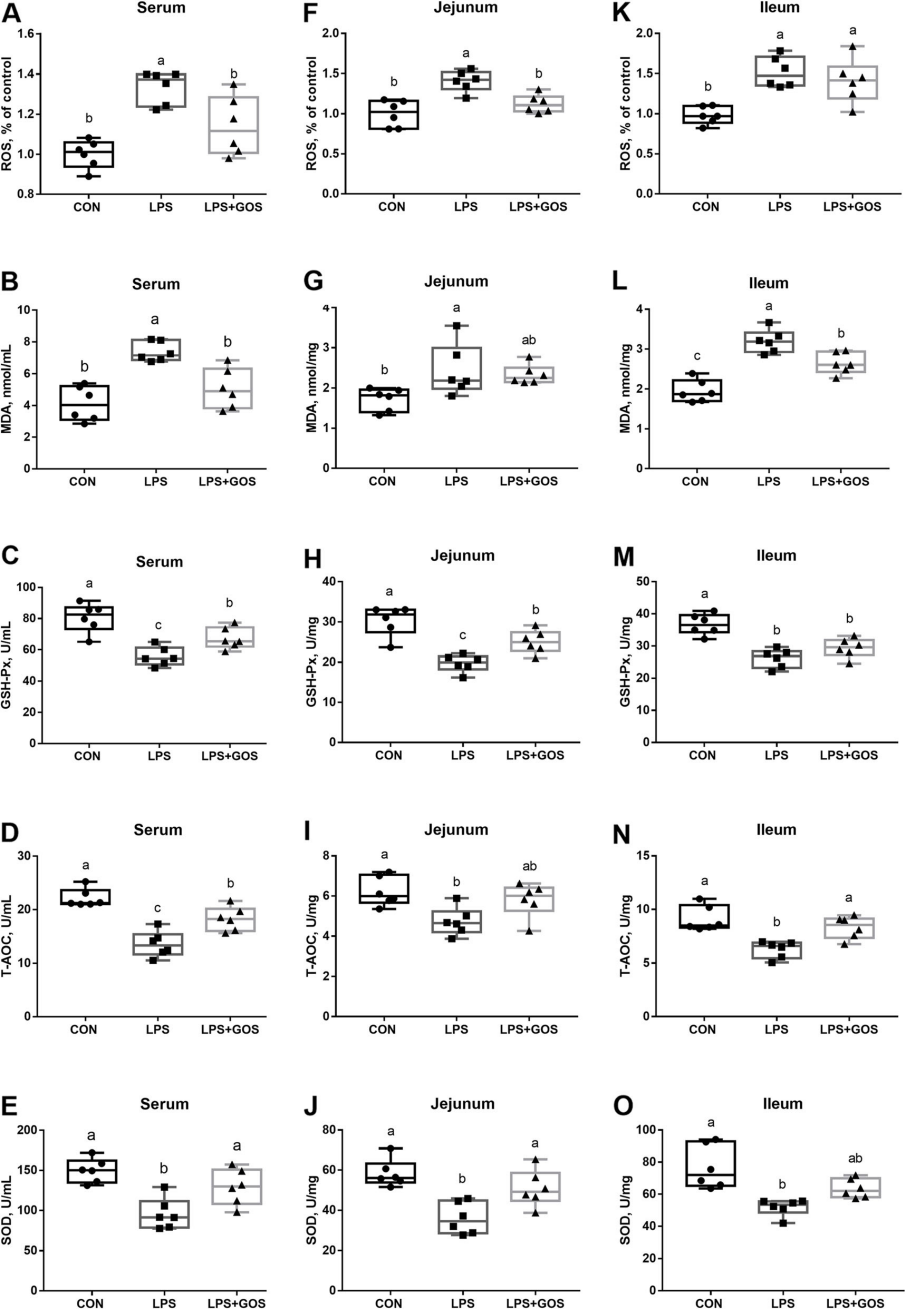
Effects of early-life galacto-oligosaccharides (GOS) supplement on antioxidative indexes in serum and intestine of LPS-challenged piglets. Piglets were assigned and treated using the same condition as Fig. 1. **A**-**E** ROS, MDA, GSH-Px, T-AOC and SOD level in serum, (**F**-**J**) ROS, MDA, GSH-Px, T-AOC and SOD level in jejunum, (**K**-**O**) ROS, MDA, GSH-Px, T-AOC and SOD level in ileum of suckling piglets. A significant difference (*P* < 0.05) among different groups is indicated by different letters. Data are expressed as means ± SD, *n* = 6

As shown in Fig. 2, the levels of ROS and MDA in serum, jejunum and ileum in LPS group were higher (*P* < 0.05) than those in CON group. The activities of GSH-Px, T-AOC and SOD (*P* < 0.05) in serum, jejunum and ileum in LPS group were lower than those in CON group. However, GOS could significantly reduce the LPS-induced increment of ROS level in serum and jejunum, and MDA level in serum and ileum (*P* < 0.05), inhibited the reduced GSH-Px, T-AOC and SOD activities in serum, GSH-Px and SOD activities in jejunum and T-AOC activity in ileum (*P* < 0.05).

### Intestinal morphology

**Fig. 3:**
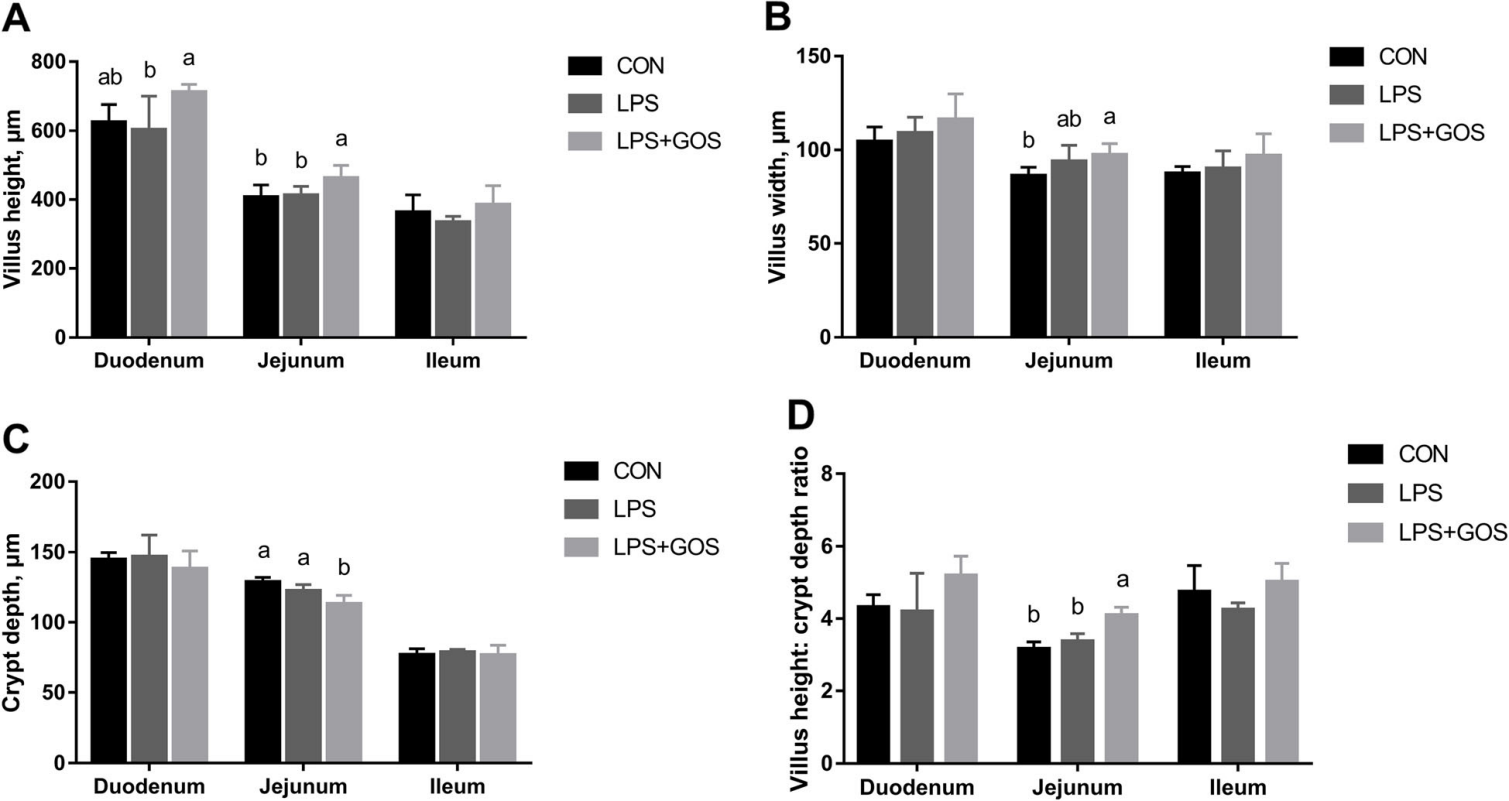
Effects of early-life galacto-oligosaccharides (GOS) supplement on intestinal morphology of LPS-challenged piglets. Piglets were assigned and treated using the same condition as Fig. 1. **A** Villus height, **B** Villus width, **C** Crypt depth and **D** Villus height:crypt depth radio of small intestinal morphology in LPS-challenged piglets. A significant difference (*P* < 0.05) among different groups is indicated by different letters. Data are expressed as means ± SD, *n* = 6

Figure 3 shows the results of the intestinal morphology according to the type of experimental treatments and small intestinal segment. Histomorphological differences were observed in the jejunum between the LPS + GOS group and the CON group or LPS group as the villus height, and the villus height/crypt depth were increased, while the crypt depth was decreased. In addition, the duodenum of piglets supplemented with GOS for 13 d had a higher villus height than that in LPS group. No significant differences were observed between CON group and LPS group.

### Intestinal digestion and absorption

**Fig. 4:**
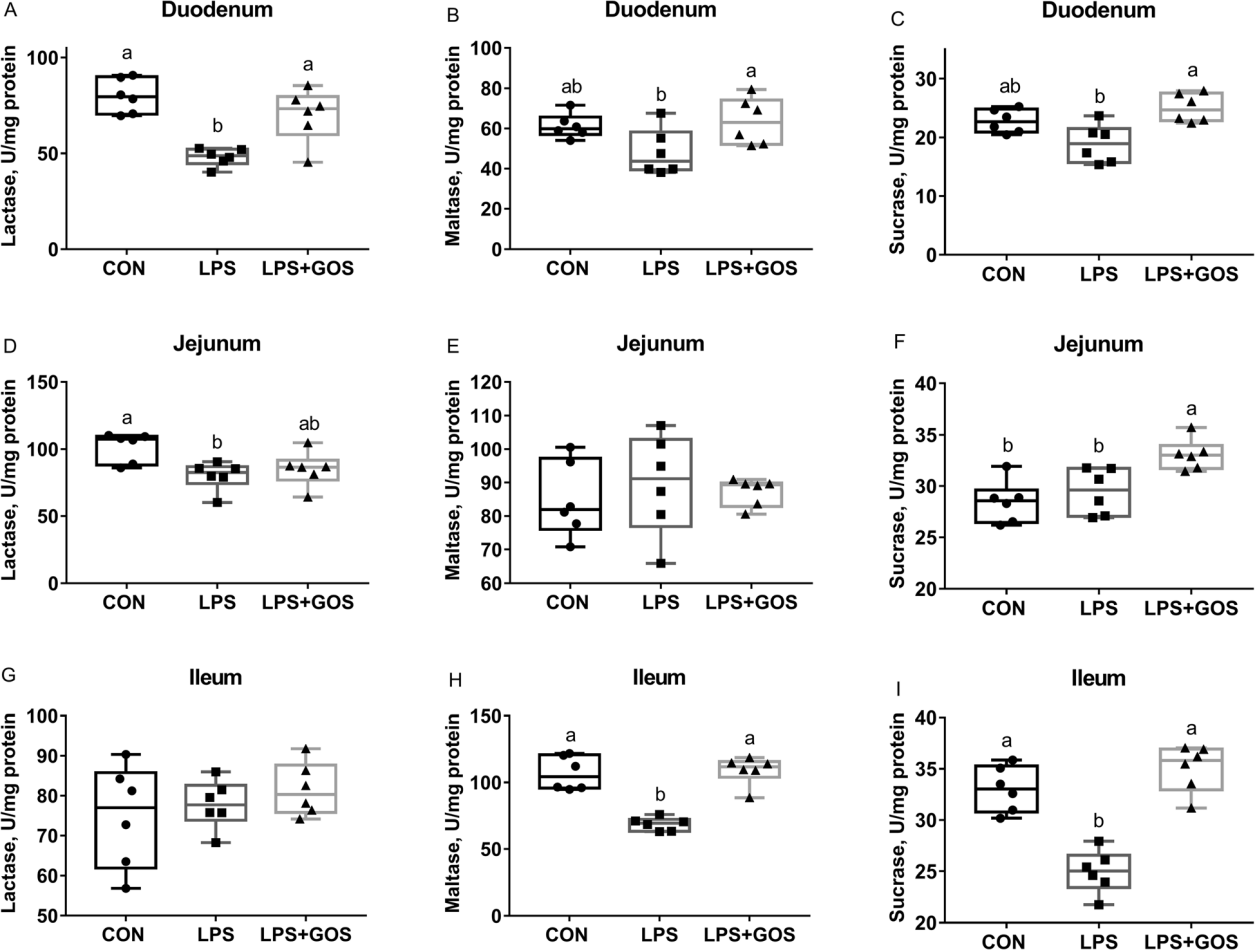
Effects of early-life galacto-oligosaccharides (GOS) supplement on disaccharidase activity in small intestinal mucosa of LPS-challenged piglets. Piglets were assigned and treated using the same condition as Fig. 1. **A**-**C** The lactase, maltase and sucrase activity in duodenum of LPS-challenged piglets. **D**-**F** The lactase, maltase and sucrase activity in jejunum of LPS-challenged piglets. **G**-**I** The lactase, maltase and sucrase activity in ileum of LPS-challenged piglets. A significant difference (*P* < 0.05) among different groups is indicated by different letters. Data are expressed as means ± SD, *n* = 6

**Fig. 5:**
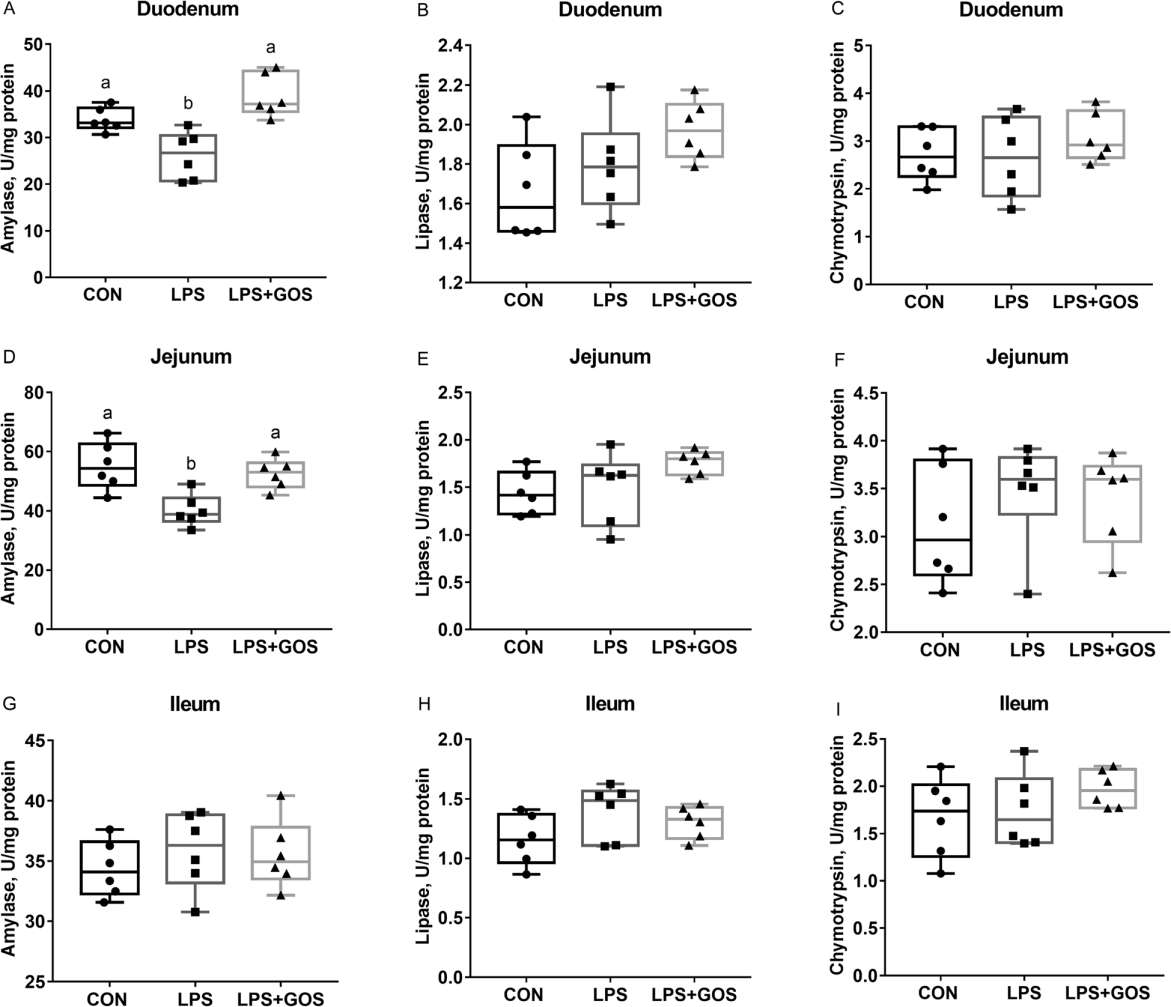
Effects of early-life galacto-oligosaccharides (GOS) supplement on digestive enzyme activity in small intestinal content of LPS-challenged piglets. Piglets were assigned and treated using the same condition as Fig. 1. **A**-**C** The amylase, lipase and chymotrypsin activity in duodenal content of LPS-challenged piglets. **D**-**F** The amylase, lipase and chymotrypsin activity in jejunal content of LPS-challenged piglets. **G**-**I** The amylase, lipase and chymotrypsin activity in ileal content of LPS-challenged piglets. A significant difference (*P* < 0.05) among different groups is indicated by different letters. Data are expressed as means ± SD, *n* = 6

Disaccharidases enzyme activities in brush border are shown in Fig. 4. The LPS challenge significantly reduced the activities of duodenal and jejunal lactase, ileal maltase and sucrase (*P* < 0.05). GOS supplement for 13 d attenuated the LPS-induced decrease of duodenal lactase (*P* < 0.05), ileal maltase and sucrase (*P* < 0.05). The digestive enzyme activities in intestinal content are shown in Fig. 5. The LPS challenge significantly reduced the activities of duodenal and jejunal amylase (*P* < 0.05). Supplementing with GOS for 13 d attenuated the LPS-induced decrease of duodenal and jejunal amylase (*P* < 0.05).

**Fig. 6:**
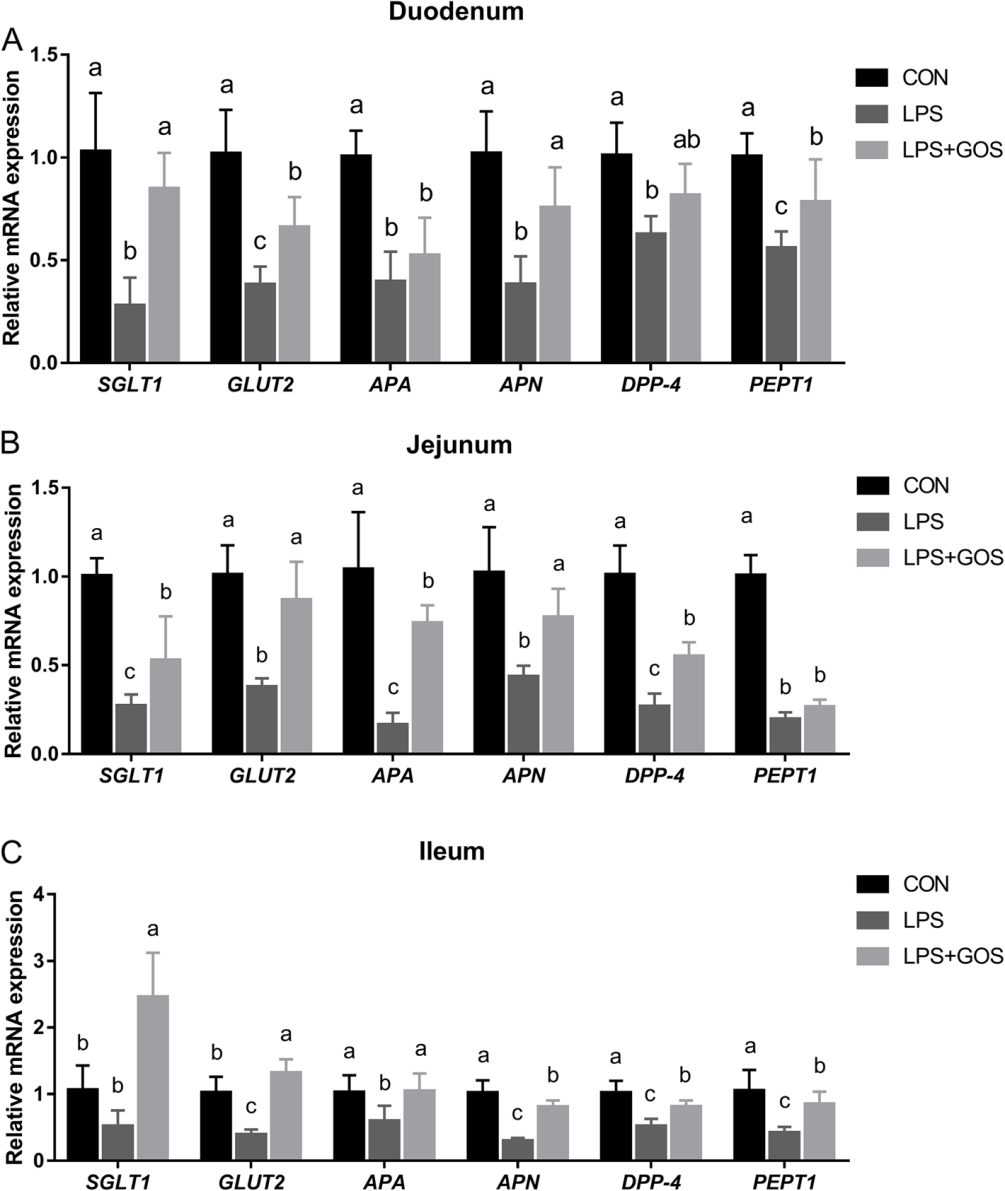
Effects of early-life galacto-oligosaccharides (GOS) supplement on mRNA expression of genes related to nutrient digestion and absorption in small intestinal mucosa of LPS-challenged piglets. Piglets were assigned and treated using the same condition as Fig. 1. **A** The gene expression of *SGLT1*, *GLUT2*, *APA*, *APN*, *DPP-4* and *PEPT1* in duodenal mucosa of LPS-challenged piglets. **B** The gene expression of *SGLT1*, *GLUT2*, *APA*, *APN*, *DPP-4* and *PEPT1* in jejunal mucosa of LPS-challenged piglets. **C** The gene expression of *SGLT1*, *GLUT2*, *APA*, *APN*, *DPP-4* and *PEPT1* in ileal mucosa of LPS-challenged piglets. A significant difference (*P* < 0.05) among different groups is indicated by different letters. Data are expressed as means ± SD, *n* = 6

The expression of nutrient-absorbing genes (sodium-dependent glucose transporter 1, *SGLT1*; glucose transporter 2, *GLUT2*; peptide transporter 1, *PEPT1*) and peptidase-digesting genes (aminopeptidase A, *APA*; aminopeptidase N, *APN*; dipeptidyl peptidase 4, *DPP-4*) are shown in Fig. 6. The mRNA expressions of *GLUT2*, *APA*, *APN*, *DPP-4* and *PEPT1* were affected by LPS challenge in duodenum, jejunum, and ileum (*P* < 0.05), and the mRNA expression of *SGLT1* was affected by LPS challenge only in duodenum and jejunum (*P* < 0.05). Moreover, the mRNA expression of *SGLT1*, *GLUT2, APN* and *PEPT1* in duodenum, *SGLT1*, *GLUT2*, *APA*, *APN* and *DPP-4* in jejunum, and *GLUT2*, *APA*, *APN*, *DPP-4* and *PEPT1* in ileum of piglets supplemented with GOS were significantly higher than those of LPS group (*P* < 0.05).

### Intestinal barrier integrity

As shown in Table 2, the LPS challenge significantly increased the levels of plasma DAO, and reduced the activities of duodenal, jejunal and ileal DAO (*P* < 0.05). The GOS supplement mitigated the LPS-induced increment of plasma DAO and reduction of duodenal, jejunal and ileal DAO activities (*P* < 0.05).

**Table 2:** Effects of galacto-oligosaccharides (GOS) on diamine oxidase (DAO) in LPS-challenged piglets (*n* = 6)

	CON	LPS	LPS + GOS	*P* value
Plasma DAO, units/mL	13.11 ± 0.85^c^	19.82 ± 2.11^a^	16.77 ± 1.95^b^	< 0.001
Duodenal mucosa DAO, units/mg protein	5.61 ± 0.63^a^	4.31 ± 0.69^b^	4.80 ± 0.70^ab^	0.015
Jejunal mucosa DAO, units/mg protein	6.69 ± 0.83^a^	4.52 ± 0.31^b^	5.84 ± 0.59^a^	< 0.001
Ileal mucosa DAO, units/mg protein	7.74 ± 0.40^a^	5.92 ± 1.11^b^	7.42 ± 0.78^a^	0.003

^a-c^Values in the same row with different superscripts are significantly different (*P* < 0.05)

**Fig. 7:**
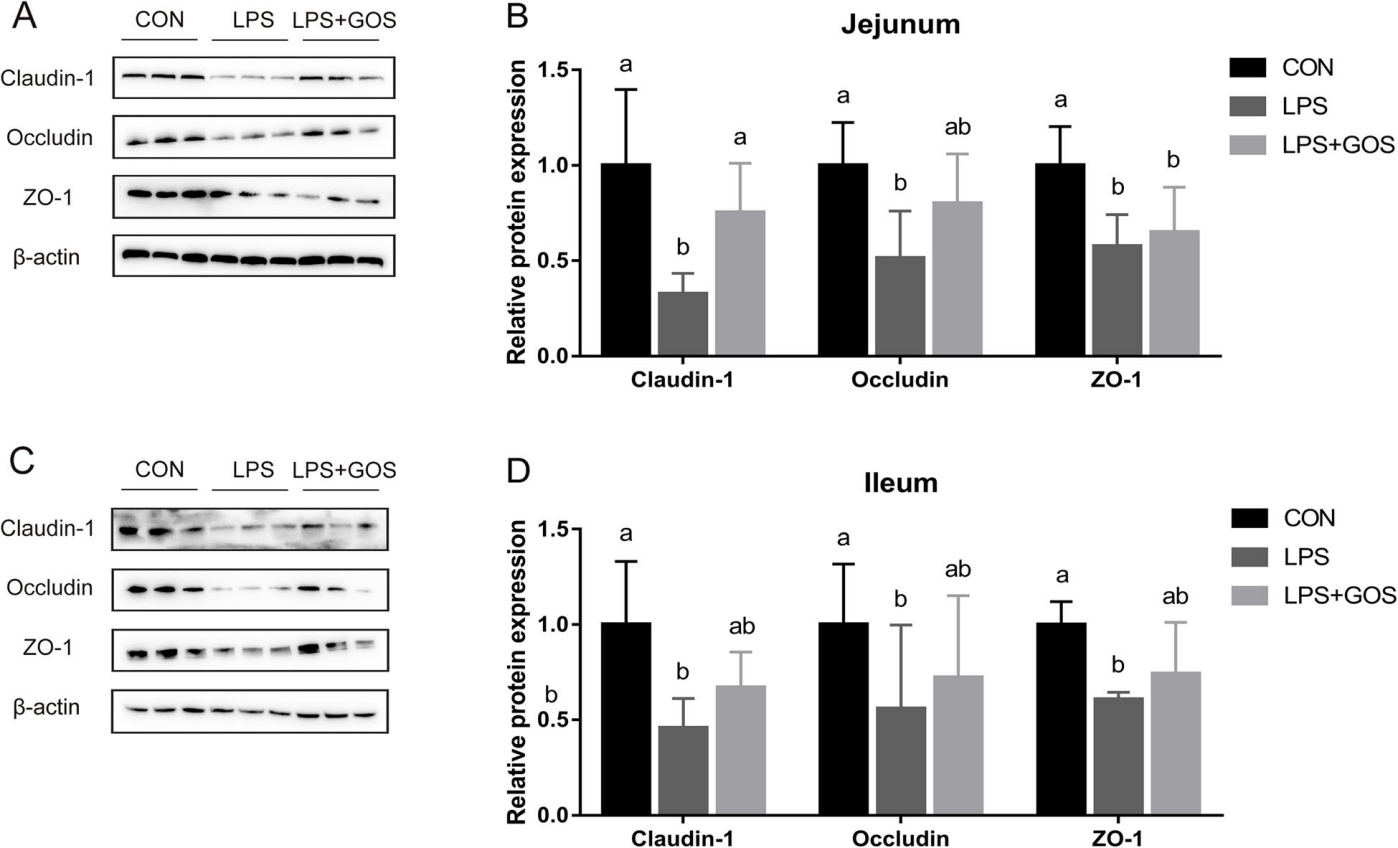
Effects of early-life galacto-oligosaccharides (GOS) supplement on relative protein expression of the jejunal and ileal tight junction in suckling piglets. Piglets were assigned and treated using the same condition as Fig. 1. **A**-**B** The relative protein expression of ZO-1, Claudin-1 and Occludin in jejunum of suckling piglets. **C**-**D** The relative protein expression of ZO-1, Claudin-1 and Occludin in ileum of suckling piglets. A significant difference (*P* < 0.05) among different groups is indicated by different letters. Data are expressed as means ± SD, *n* = 6

Figure 7 shows the protein expressions of ZO-1, Occludin and Claudin-1 in the jejunum and ileum of piglets. LPS challenge significantly decreased the protein expressions of ZO-1, Occludin and Claudin-1 in jejunum and ileum (*P* < 0.05). GOS treatment relieved the LPS-induced reduction of jejunal Claudin-1 protein expression (*P* < 0.05). In addition, GOS treatment also attenuated the LPS-induced decrease of the protein expression of Occludin in jejunum, and ZO-1, Occludin and Claudin-1 in ileum. However, there was no significant difference between LPS group and LPS + GOS group.

### Intestinal apoptosis

**Fig. 8:**
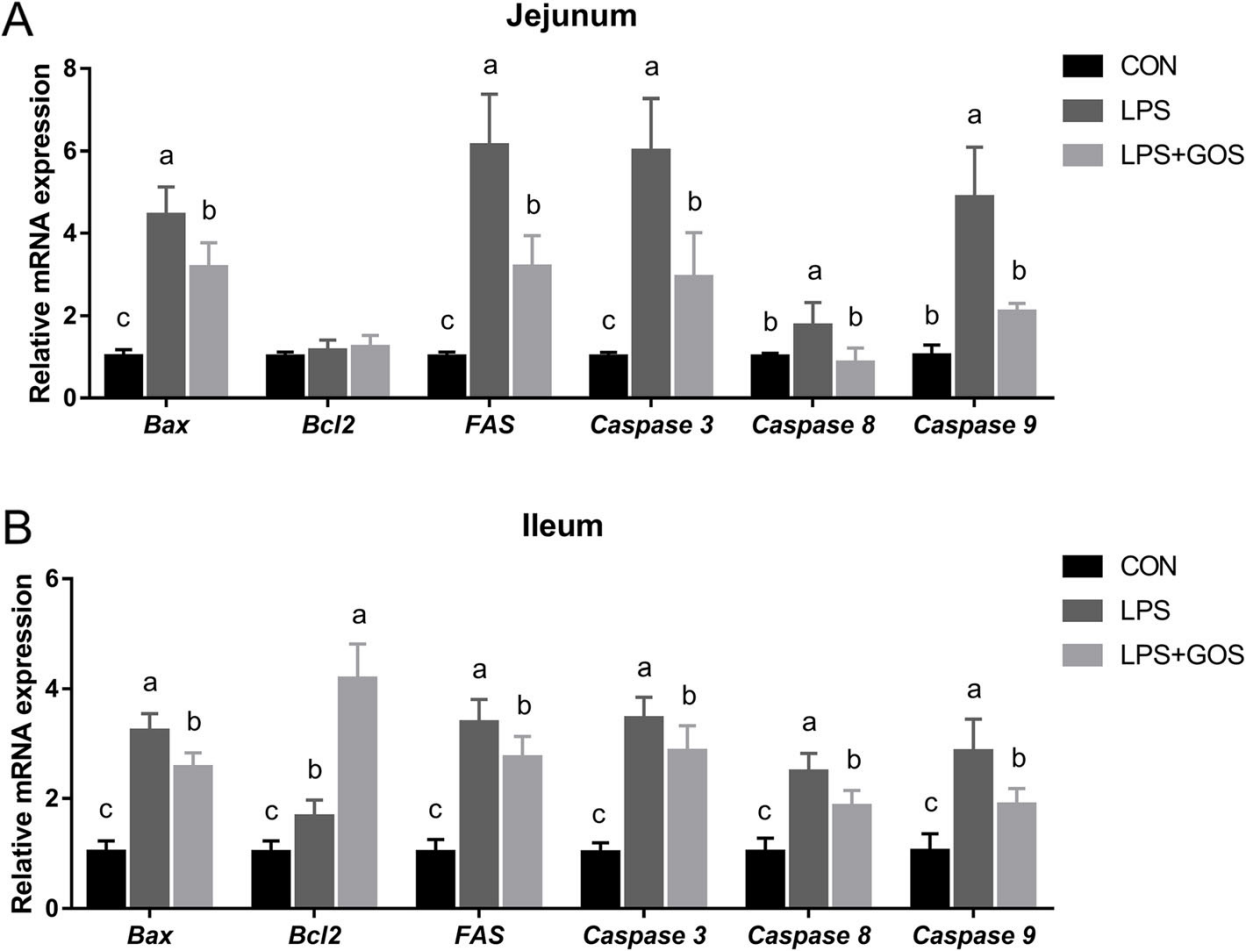
Effects of early-life galacto-oligosaccharides (GOS) supplement on apoptosis-related gene expression in jejunum and ileum of LPS-challenged piglets. Piglets were assigned and treated using the same condition as Fig. 1. **A** The relative mRNA expression of *Bax*, *Bcl2*, *FAS*, *Caspase 3*, *Caspase 8* and *Caspase 9* in jejunum of suckling piglets. **B** The relative mRNA expression of *Bax*, *Bcl2*, *FAS*, *Caspase 3*, *Caspase 8* and *Caspase 9* in ileum of suckling piglets. The value of protein expression was the ratio of the densitometry units of tight junction protein to β-actin. A significant difference (*P* < 0.05) among different groups is indicated by different letters. Data are expressed as means ± SD, *n* = 6

The mRNA expressions of apoptosis-related genes in jejunum and ileum are illustrated in Fig. 8. Compared to the CON group, LPS challenge increased the pro-apoptotic factor B-cell lymphoma-2-associated X protein (*Bax*), Fas cell surface death receptor (*FAS*), cysteinyl aspartate-specific proteinase-3 (*Caspase 3*), cysteinyl aspartate-specific proteinase-8 (*Caspase 8*) and cysteinyl aspartate-specific proteinase-9 (*Caspase 9*) mRNA expressions in jejunum and ileum (*P* < 0.05), and increased the anti-apoptotic factor B-cell lymphoma-2 (*Bcl2*) mRNA expression in ileum (*P* < 0.05). However, GOS treatment inhibited the LPS-induced increase of *Bax*, *FAS*, *Caspase 3*, *Caspase 8* and *Caspase 9* mRNA expressions in jejunum and ileum (*P* < 0.05). In addition, GOS treatment also significantly increased *Bcl2* mRNA expression in ileum when compared with the CON or LPS group (*P* < 0.05).

### Intestinal HO-1, NQO-1 and p-AMPK expression

**Fig. 9:**
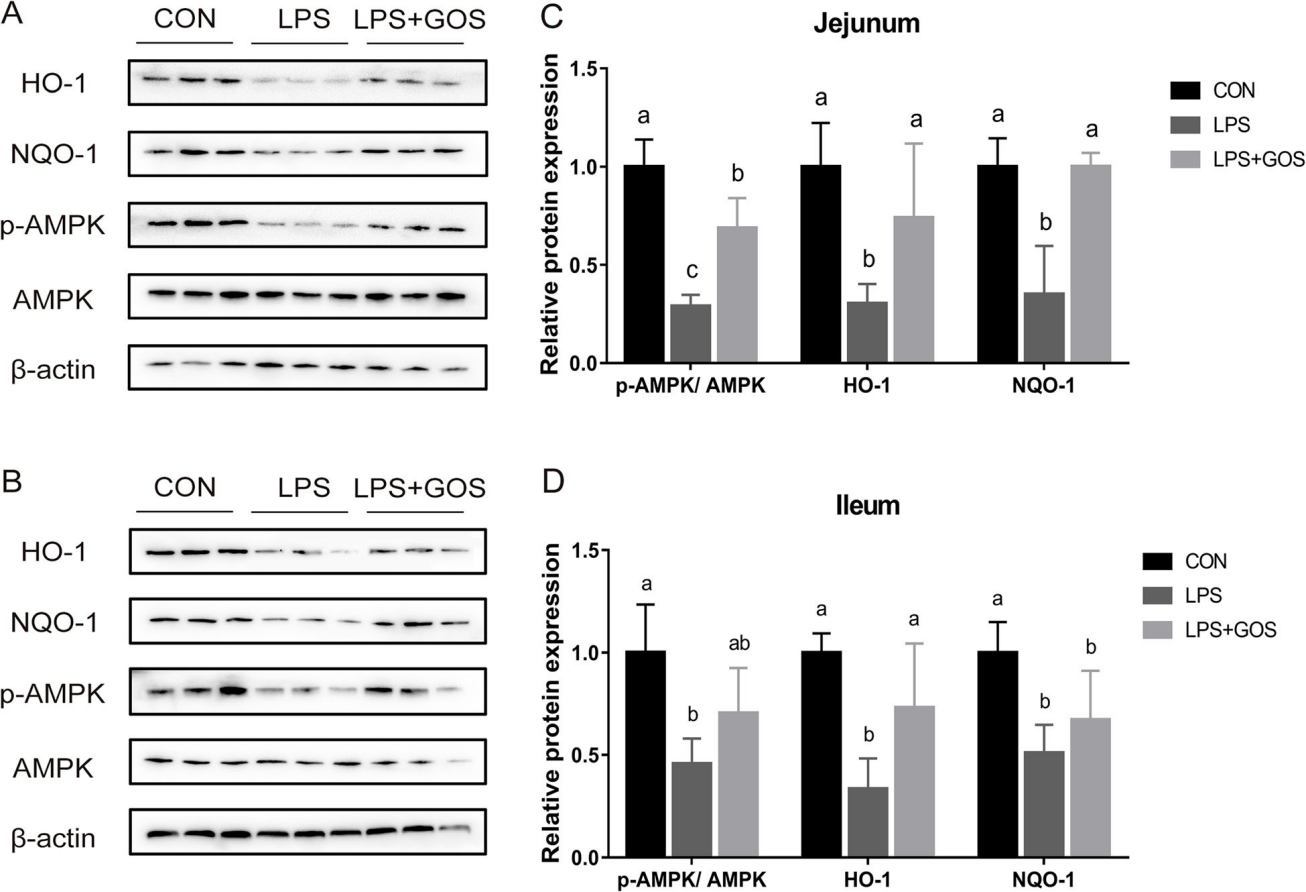
Effects of early-life galacto-oligosaccharides (GOS) supplement on HO-1, NQO-1 and p-AMPK level in jejunum and ileum of LPS-challenged piglets. Piglets were assigned and treated using the same condition as Fig. 1. **A**-**B** The blots of HO-1, NQO-1, p-AMPK, AMPK and β-actin in the jejunal and ileal mucosa of LPS-challenged piglets. **C**-**D** The relative protein expressions of HO-1, NQO-1 and p-AMPK/AMPK in jejunum and ileum of suckling piglets. A significant difference (*P* < 0.05) among different groups is indicated by different letters. Data are expressed as means ± SD, *n* = 6

To understand the underlying mechanism of the protective effect of the early life GOS supplement on suckling piglets challenged by LPS, we investigated the relative protein expression of HO-1, NQO-1 and p-AMPK. As shown in Fig. 9, LPS induced a decrease of the relative protein expression of HO-1, NQO-1 and p-AMPK (*P* < 0.05) in jejunum and ileum, but the early life GOS supplement suppressed the LPS-induced decrease of HO-1, NQO-1 and p-AMPK level (*P* < 0.05) in jejunum, and repressed the LPS-induced decrease of HO-1 level (*P* < 0.05) in ileum.

## Discussion

There is a growing research interest in understanding the effects of prebiotic on antioxidant capacity and barrier function. Prebiotics such as GOS have been reported to enhance the intestinal barrier function [27, 28] and antioxidant capacity [11, 29, 30]. A progressive oxidation shift in the glutathione and glutathione disulfide redox status caused by oxidative stress induces abnormal proliferation, growth stagnation, differentiation and apoptosis which cause the intestinal damage and injury of gut barrier, leading to serious inflammatory bowel disease and colon cancer [31–33]. In addition, LPS challenge has been shown to disrupt cellular redox homeostasis and tight junction assembly, leading to the reduced intestinal barrier function in weaned piglets [16]. Accordingly, we evaluated the protective effect of the early-life GOS supplement on antioxidant ability and intestinal integrity after LPS challenge in a suckling piglet model. Our present study demonstrated that the early-life GOS supplement affected ADG of suckling piglets. Similar observations were made by Xing et al. [11] and Wu et al. [34], indicating the growth-enhancing benefits of GOS in piglets. We also found that GOS alleviated the oxidative stress by stimulating antioxidant enzymes production, which was related to AMPK signaling pathway, thereby maintaining gut function homeostasis.

In general, after LPS stimulation, phagocytes are induced to produce excessive ROS, leading to the imbalance between ROS and antioxidants [35]. In our study, LPS challenge induced excessive the ROS release and MDA production, and decreased the activities of GSH-Px, SOD and T-AOC. These results were consistent with a recent report about using LPS to establish a cell oxidative injury model to study the protective effects of epidermal growth factor on IPEC-J2 cells [36]. Previous studies have reported that GOS have an antioxidant capacity [11, 30]. Consistent with these studies, the increase of ROS release and MDA production and the decrease of antioxidant enzyme levels induced by LPS were significantly blocked by GOS in present study, suggesting that GOS alleviated LPS-induced oxidative stress. In addition, Lan et al. reported that H_2_O_2_ decreased the villus height and villus height to crypt depth ratio of the small intestine in rats, which indicated that the oxidative stress damage was accompanied by the change of intestinal morphology [37]. However, the intestinal morphology had no significant changes after LPS challenge in present study, which was different from the results reported by Xiao et al. where the dynamic effect of LPS challenge on intestinal injury was suggested in a piglet model [38]. This difference was probably due to the lower administering dose of LPS in our study compared to that in the research reported by Xiao et al. Imbalances of ROS caused mitochondria injury, leading to the precipitous reduce of ATP concentrations and the disruption of ions homeostasis [39]. A previous report has shown that the nutrient transporters functions depend on ions such as Na^+^ and H^+^ [40]. These researches suggested that oxidative stress might cause the intestinal absorption and digestion function disorder. In our study, the piglets with LPS challenge had lower mRNA expressions of nutrient-absorbing genes and peptidase-digesting genes, and lower disaccharidases enzyme activities in small intestine. These results were consistent with the recent reports which demonstrated intestinal digestive dysfunction in an LPS challenged piglet model [41] and the dynamic changes of mucosal enzyme activity in an *E.coli* challenged piglet model [42]. Moreover, the reduction of the disaccharidases enzyme activities and the mRNA expressions of nutrient-absorbing and peptidase-digesting genes induced by LPS were significantly blocked by GOS in present study, implying that GOS alleviated LPS-induced intestinal absorption and digestion function disorder. Overall, LPS challenge induced oxidative stress, leading to the intestinal absorption and digestion function disorder; while GOS alleviated the oxidative stress, and intestinal absorption and digestion disorder caused by LPS challenge.

Intestinal oxidative stress is one of the activators causing intestinal barrier dysfunction [33]. Consistent with the results of LPS-induced change in intestinal oxidative status, LPS challenge damaged intestinal integrity accompanied with the increased level of plasma DAO and the decreased level of intestinal DAO. These results suggested that the intestinal barrier dysfunction also arose with the occurrence of LPS-induced intestinal oxidative stress. The GOS treatment was able to attenuate the LPS-induced intestinal integrity damage evidenced by the reduced plasma DAO level as well as the increased intestinal DAO level. Tight junctions have a critical role in the maintaining of intestinal barrier function, and the depletion of tight junction expression disrupts the ability of intestinal integrity [43]. Consistent with the changes of intestinal integrity, LPS challenge indeed reduced the expression of tight junctions. Previous studies have indicated that oligosaccharides decreased intestinal permeability and facilitated the assembly and expression of tight junctions [28, 44]. We previously discovered that GOS contributed to improving the tight junction expression in suckling piglets under a normal condition [45]. In present study, we further revealed that GOS had a protective effect on intestinal barrier dysfunction caused by LPS challenge. Therefore, the supplementation with GOS could be a potential nutritional approach to alleviate the intestinal barrier dysfunction in LPS-challenged piglets.

Intestinal oxidative stress led to growth stagnation, differentiation and apoptosis, intestinal cells damage and intestinal barrier injury [33]. Sharifi et al. reported that LPS increased the protein expression of Bax and Caspase 3 through the mitochondrial pathway, leading to apoptosis and even death in PC12 cells [46]. In addition, Tang et al. reported that LPS also increased the mRNA expression of *FAS* through the death receptor pathway leading to apoptosis in IPEC-J2 cells [36]. Consistent with these results, our results showed that LPS induced the increased apoptosis-related gene transcriptional levels, including *Bax*, *Fas*, *Caspase 3*, *Caspase 8* and *Caspase 9*, suggesting that LPS induced intestinal cell apoptosis through a mitochondrial pathway and death receptor pathway. Previous study have shown that GOS increased the protein expression of Bcl2 and decreased the protein expression of Bax in the spinal cord of mice [47]. Consistent with its results, in this study, the early-life GOS treatment could inhibit the cell apoptotic caused by LPS challenge. Moreover, the anti-apoptotic factor *Bcl2* mRNA abundance was increased in both LPS group and LPS + GOS group in ileum, but the increased amplitude in LPS + GOS group was greater than that in LPS group. It suggested that the anti-apoptotic process of the body was triggered by the LPS challenge, and the early life GOS supplement could enhance this anti-apoptotic effect. Overall, these results indicated that the early life GOS supplement could enhance the anti-apoptotic factor *Bcl2* mRNA abundance to repress the apoptosis-related gene transcriptional expressions, and maintain the integrity of the epithelial barrier.

Nrf2 is regarded as a pivotal nuclear transcription factor that effectively promotes endogenous antioxidant enzyme gene transcription. HO-1 and NQO-1 are critical components of the cellular defense against oxidative stress, and their expression levels are regulated by Nrf2 [19]. Previous studies demonstrated that LPS elevated ROS and MDA levels in cells and tissues, and directly inhibited the expression of antioxidant enzymes by inhibiting Nrf2 signaling [48, 49]. Consist with these findings, we found that the generation of ROS triggered by LPS challenge suppressed the expression of HO-1 and NQO-1, while the early-life GOS supplement effectively inhibited the accumulation of ROS and promoted the expression of HO-1 and NQO-1. In addition, AMKP is an important kinase that regulates Nrf2 activity. When AMPK is activated, Nrf2 activity is also increased; conversely, the inactivation of AMPK results in the Nrf2 downregulation [50]. The facts that AMPK activation mediated Nrf2 activity and elevated HO-1 and NQO1 expressions have been demonstrated in many experiments including AMPK knockout mouse embryonic fibroblasts [51], a Pb-exposed rat model [52], human endothelial cells [53]. LPS-stimulated macrophages [20], and LPS-stimulated microglia [22]. Vitali et al. have shown that the oxidative stress caused by LPS was able to inhibit AMPK phosphorylation [54]. Consistent with this result, we found that LPS challenge reduced p-AMPK level in small intestinal mucosa of suckling piglets. Interestingly, we found that the early-life GOS supplement blocked the reduction of p-AMPK induced by the LPS challenge. Previous studies have presented that GOS can be degraded by gut microbes to produce butyrate in suckling piglets [8, 55]. Previous studies have also suggested that butyrate can act as an endogenous agonist of AMPK [45]. Thus, it is a logical speculation that gut microbiota derived butyrate mediates GOS activation of AMPK signaling in intestinal mucosa of suckling piglets, and these changes alleviate the inhibitory effect of LPS on AMPK signaling. However, the mechanism of GOS activating AMPK still needs a further investigation.

In summary, the early-life GOS supplement activates the AMPK signaling pathway, and attenuates LPS-induced intestinal oxidative stress through down-regulating the production of ROS and MDA, and up-regulating the antioxidant enzymes activity in suckling piglets.

## Supplementary Information

**Additional file 1: Table S1.** Primer sequences for quantitative real-time PCR analysis

## Data Availability

The datasets used during the current study available from the corresponding author on reasonable request.

## Abbreviations

ADG: Average daily gain
APA: Aminopeptidase A
APN: Aminopeptidase N
AMPK: AMP-activated protein kinase
Bcl2: B-cell lymphoma-2
Bax: B-cell lymphoma-2-associated X protein
BCA: Bicinchoninic acid
Caspase 3: Cysteinyl aspartate-specific proteinase-3
Caspase 8: Cysteinyl aspartate-specific proteinase-8
Caspase 9: Cysteinyl aspartate-specific proteinase-9
DAO: Diamine oxidase
DPP-4: Dipeptidyl peptidase 4
FAS: Fas cell surface death receptor
GOS: Galacto-oligosaccharides
GLUT2: Glucose transporter 2
GSH-Px: Glutathione peroxidase
HE: Hematoxylin and Eosin
HO-1: Hemeoxygenase-1
LPS: Lipopolysaccharide
MDA: Malondialdehyde
NQO-1: NAD(P)H/quinone acceptor oxidoreductase-1
Nrf2: Nuclear factor (erythroid-derived-2)-like 2
ROS: Reactive oxygen species
PEPT1: Peptide transporter 1
PVDF: Polyvinylidene difluoride
SI: Small intestine
SGLT1: Sodium-dependent glucose transporter 1
SDS–PAGE: Sodium dodecyl sulfate–polyacrylamide gel electrophoresis
SOD: Superoxide dismutase
TBA: Thiobarbituric acid
T-AOC: Total anti-oxidation capacity
ZO-1: zonula occludens-1
