# Supplementary material for: Early-life galacto-oligosaccharides supplementation alleviates the small intestinal oxidative stress and dysfunction of lipopolysaccharide-challenged suckling piglets
Source: J Anim Sci Biotechnol. 2022 Jun 3;13:70. doi: 10.1186/s40104-022-00711-5 (PMC9164537; doi:10.1186/s40104-022-00711-5)
Supplement: Supplementary file 1 — Additional file 1: Table S1. Primer sequences for quantitative real-time PCR analysis [file 40104_2022_711_MOESM1_ESM.docx]

**Table S1** **Primer sequences for quantitative real-time PCR analysis.**

| Gene^a^ | Nucleotide sequences 5’-3’ |
| --- | --- |
| *SGLT1* | CCACTTTCCCTATAAAACCTCAC/CTCCATCAAACTTCCATCCTCAG |
| *GLUT2* | CCTGCTTGGTCTATCTGCTGTG/TTGATGCTTCTTCCCTTTCTTT |
| *APA* | GTCTCTACCACCTGACGAT/CTCTGTAAGTGATGAGTCC |
| *APN* | ACATCACTCTCATCCACCCT/GCAATCACAGTGACAACTCG |
| *DPP-4* | CCTCCGGCGTCTGTGTTA/TGGATTCAGCTCACAGCT |
| *PEPT1* | GATGAAATGTGAGCGTATGGG/AAAGAGGGAGGATCTGGAAAA |
| *Bax* | CTGACGGCAACTTCAACTGG/CGTCCCAAAGTAGGAGAGGA |
| *Bcl2* | AGCATGCGGCCTCTATTTGA/GGCCCGTGGACTTCACTTAT |
| *FAS* | TGATGCCCAAGTGACTGACC/GCAGAATTGACCCTCACGAT |
| *Caspase 3* | GTGGGACTGAAGATGACA/ACCCGAGTAAGAATGTG |
| *Caspase 8* | GGATCCCAGGATTTGCCTCC/AGGATGGCCCTCTTCTCCAT |
| *Caspase 9* | AATGCCGATTTGGCTTACGT/CATTTGCTTGGCAGTCAGGTT |
| *GAPDH* | ATGGTGAAGGTCGGAGTGAAC/CTCGCTCCTGGAAGATGGT |

*^a^SGLT1*: Sodium glucose co-transporter 1; *GLUT2*: Glucose transporter type 2; *APA*: Aminopeptidase A; *APN*: Aminopeptidase N; *DPP-4*: Dipeptidyl peptidase-4; *PEPT1*: Peptide transporter 1; *Bax*: B-cell lymphoma-2-associated X protein; *Bcl2*: B-cell lymphoma-2; *FAS*: Fas cell surface death receptor; *Caspase 3*: cysteinyl aspartate-specific proteinase-3; *Caspase 8*: cysteinyl aspartate-specific proteinase-8; *Caspase 9*: cysteinyl aspartate-specific proteinase-9; *GAPDH*: Glyceraldehyde phosphate dehydrogenase.
